# Bis(3-acetyl-6-methyl-2-oxo-2*H*-pyran-4-olato)bis­(dimethyl sulfoxide)nickel(II)

**DOI:** 10.1107/S1600536809034655

**Published:** 2009-09-12

**Authors:** Amel Djedouani, Sihem Boufas, Abderrahmen Bendaas, Magali Allain, Gilles Bouet

**Affiliations:** a Laboratoire d’Electrochimie des Matériaux, Moléculaires et Complexes, Département de Génie des Procédés, Faculté des Science de l’Ingénieur, Université Farhet Abbas de Setif, DZ-19000 Sétif, Algeria; b Université 20 Aout 1955, Skikda, Algeria; c CIMM, CNRS UMR 6200, Faculté des Science, Angers Cedex, France; d SONAS, EA 921, Université D’Angers, Faculté de Pharmacie, Angers Cedex, France

## Abstract

In the title compound, [Ni(C_8_H_7_O_4_)_2_{(CH_3_)_2_SO}_2_], the Ni^II^ atom is located on a crystallographic centre of symmetry and has a distorted octa­hedral coordination geometry of type *M*O_6_. The bidentate dehydro­acetic acid (DHA) ligands occupy the equatorial plane of the complex in a *trans* configuration, and the dimethyl sulfoxide (DMSO) ligands are weakly coordinated through their O atoms in the axial positions.

## Related literature

3-Acetyl-4-hydr­oxy-6-methyl-2-oxo-2*H*-pyran (dehydro­acetic acid) (Arndt *et al.*, 1936 ▶) is a versatile starting material for the synthesis of a wide variety of heterocyclic ring systems (Tan & Ang, 1988 ▶). It has been shown to possess modest anti­fungal properties, see: Rao *et al.* (1978 ▶). For natural fungicides possessing structures analogous to 5,6-dihydro­dehydroacetic acid, see: Bartels-Keith (1960 ▶); Miyakado *et al.* (1982 ▶); Ayer *et al.* (1988 ▶). The complexes of DHA with copper and with several other transition metal cations are fungistatic, see: Rao *et al.* (1978 ▶). For the nickel–DHA complex, see: Casabò *et al.* (1987 ▶). The configuration of the complex mol­ecule is similar to that found in [Zn(DHA)_2_·2(DMSO) and Cd(DHA)_2_·2(DMSO)] (Zucolotto Chalaça *et al.*, 2002 ▶), [Cu(DHA)_2_·2(DMSO)] (Djedouani *et al.*, 2006 ▶) and bis­(4,6-dibromo-2-formyl­phenolato-κ^2^ *O*,*O*′)-bis­(dimethyl sulfoxide)nickel(II) (Zhang *et al.*, 2007 ▶). For Ni—O_DMSO_ distances in similar structures, see: Ma *et al.* (2003 ▶); Tahir *et al.* (2007 ▶); Zhang *et al.* (2007 ▶).
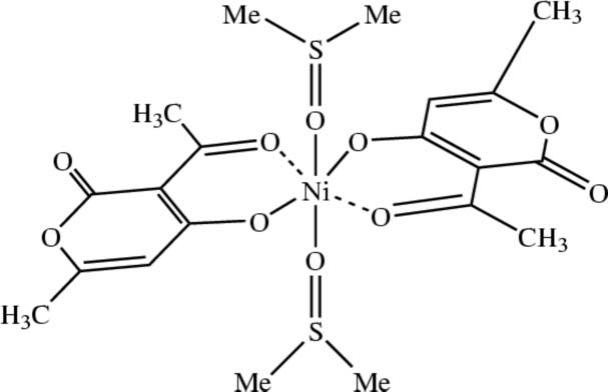


## Experimental

### 

#### Crystal data

[Ni(C_8_H_7_O_4_)_2_(C_2_H_6_OS)_2_]
*M* *_r_* = 549.24
Monoclinic, 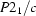

*a* = 11.3850 (10) Å
*b* = 6.2833 (4) Å
*c* = 19.7434 (15) Å
β = 123.525 (6)°
*V* = 1177.40 (16) Å^3^
*Z* = 2
Mo *K*α radiation
μ = 1.05 mm^−1^
*T* = 100 K
0.25 × 0.15 × 0.10 mm

#### Data collection

Nonius KappaCCD diffractometer
Absorption correction: multi-scan (*SADABS*; Sheldrick, 1996 ▶)  *T*_min_ = 0.902, *T*_max_ = 0.902
14084 measured reflections
2628 independent reflections
1962 reflections with *I* > 2σ(*I*)
*R*_int_ = 0.071

#### Refinement

*R*[*F* ^2^ > 2σ(*F* ^2^)] = 0.037
*wR*(*F* ^2^) = 0.098
*S* = 1.11
2628 reflections
156 parameters
H-atom parameters constrained
Δρ_max_ = 0.55 e Å^−3^
Δρ_min_ = −0.97 e Å^−3^

### 

Data collection: *COLLECT* (Nonius, 2002 ▶); cell refinement: *DENZO* and *SCALEPACK* (Otwinowski & Minor, 1997 ▶); data reduction: *DENZO* and *SCALEPACK* (Otwinowski & Minor, 1997 ▶); program(s) used to solve structure: *SIR92* (Altomare *et al.*, 1993 ▶); program(s) used to refine structure: *SHELXL97* (Sheldrick, 2008 ▶); molecular graphics: *DIAMOND* (Brandenburg, 1998 ▶) and *Mercury* (Macrae *et al.*, 2006 ▶); software used to prepare material for publication: *WinGX* (Farrugia, 1999 ▶) and *PARST* (Nardelli, 1995 ▶).

## Supplementary Material

Crystal structure: contains datablocks global, I. DOI: 10.1107/S1600536809034655/hg2559sup1.cif

Structure factors: contains datablocks I. DOI: 10.1107/S1600536809034655/hg2559Isup2.hkl

Additional supplementary materials:  crystallographic information; 3D view; checkCIF report

## Figures and Tables

**Table 1:** Selected bond lengths (Å)

Ni1—O2	1.9849 (16)
Ni1—O3	2.0159 (15)
Ni1—O1	2.1255 (18)

**Table 2:** Hydrogen-bond geometry (Å, °)

*D*—H⋯*A*	*D*—H	H⋯*A*	*D*⋯*A*	*D*—H⋯*A*
C1—H1*B*⋯O4^ii^	0.96	2.55	3.384 (4)	145
C2—H2*B*⋯O4^ii^	0.96	2.53	3.370 (4)	146
C2—H2*C*⋯O2^iii^	0.96	2.46	3.378 (4)	160

Symmetry codes: (ii) 
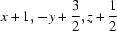
; (iii) 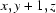.

**Fig. 1.**
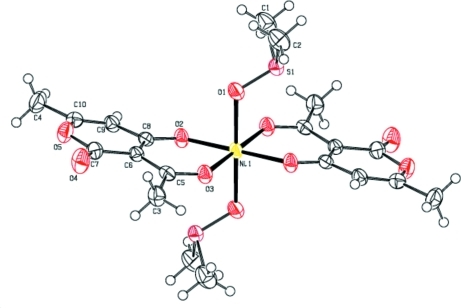
The independent components of (I), showing the atom-labelling scheme. Displacement ellipsoids are drawn at the 50% probability level.

**Fig. 2.**
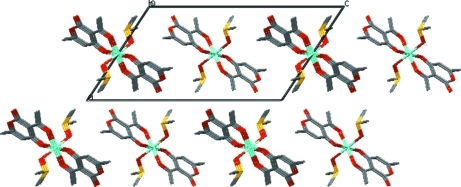
The crystal packing of (I); Hydrogen atoms have been omitted for clarity.

## supplementary crystallographic information

### Comment

3-Acetyl-4-hydroxy-6-methyl-2-oxo-2*H*-pyran (dehydroacetic acid) (Arndt *et al.*, 1936) is a versatile starting material for the synthesis of a wide variety of heterocyclic ring systems (Tan & Ang, 1988). It has been shown to possess modest antifungal properties (Rao *et al.*, 1978). The importance of similar pyrones as potential fungicides is reinforced by the existence of several natural fungicides possessing structures analogous to 5,6-dihydrodehydroacetic acid, such as alternaric acid (Bartels-Keith, 1960), the podoblastins (Miyakado *et al.*, 1982) and lachnelluloic acid (Ayer *et al.*, 1988). Also, it has been shown that the complexes of DHA with copper and with several other transition metal cations are fungistatic (Rao *et al.*, 1978). This has motivated our study of the structural characterization of complexes of dehydroacetic acid. The complex of DHA with nickel was previously reported by Casabò *et al.* (1987), but their characterization of the compound was based only on thermal and elemental analysis, and on IR and NMR spectroscopy.

We present here the crystal structure determination of the title complex, [Ni(DHA)_2_.2(DMSO)], (I) (DMSO = dimethylsulfoxide). The nature of the title compound, (I), was established by an X-ray structure determination and is shown in Fig. 1

The Ni atom lies on a crystallographic centre of symmetry with the ligands bonded to nickel in an all-*trans* fashion. The configuration of the complex molecule is similar to that found in [Zn(DHA)_2_. 2(DMSO); Cd(DHA)2.2(DMSO)] (Zucolotto Chalaça *et al.*, 2002), [Cu(DHA)_2_. 2(DMSO)] (Djedouani *et al.*,  2006), with (DHA: dehydroacetic acid) and Bis(4,6-dibromo-2-formylphenolato-κ^2^*O*,*O*')-bis(dimethyl sulfoxide)nickel(II), [Ni(C_7_H_3_Br_2_O_2_)_2_(C_2_H_6_OS)_2_] (Zhang *et al.*, 2007).

The coordination polyhedron around the Ni atom is a slightly distorted octahedron (Table 1), with the O atoms of the DMSO groups in axial positions; and the  Ni—O_DMSO_ distance is in agreement with literature values: [2.1139 (12) Å - 1.9897 (13) Å (Tahir *et al.*, 2007), 1.998 (3) Å - 2.105 (3) Å (Zhang *et al.* 2007), 2.030 (2) Å- 2.057 (2)Å (Ma *et al.*, 2003)].

The orientation of the DMSO molecule can be described by the torsion angles O3—Ni—O1—S [43.32 (4) °] and O2—Ni—O1—S [-137.70 (4) °]. The packing of (I) is stabilized by weak intermolecular C—H···O hydrogen bonds (Table 2) which form a three-dimensional network (Fig. 2).

### Experimental

Compound (I) was prepared by the reaction of dehydroacetic acid with nickel (II) chloride hexahydrate in the presence of sodium acetate (Casabò *et al.* 1987). Crystals of (I) were grown by slow evaporation of a dimethylsulfoxide solution..

### Refinement

H atoms were positioned geometrically and treated as riding, with C—H = 0.93 Å with *U*_iso_(H) = 1.2Ueq(C). The methyl H atoms were constrained to an ideal geometry (C—H = 0.96 Å) with *U*_iso_(H) = 1.2*U*_eq_(C), but were allowed to rotate freely about the C—C bonds.

### Crystal data

**Table:**

[Ni(C_8_H_7_O_4_)_2_(C_2_H_6_OS)_2_]	*F*(000) = 572
*M**_r_* = 549.24	*D*_x_ = 1.549 Mg m^−^^3^
Monoclinic, *P*2_1_/*c*	Mo *K*α radiation, λ = 0.71073 Å
Hall symbol: -P 2ybc	Cell parameters from 2190 reflections
*a* = 11.385 (1) Å	θ = 2.8–27.3°
*b* = 6.2833 (4) Å	µ = 1.05 mm^−^^1^
*c* = 19.7434 (15) Å	*T* = 100 K
β = 123.525 (6)°	Plates, colourless
*V* = 1177.40 (16) Å^3^	0.25 × 0.15 × 0.1 mm
*Z* = 2	

### Data collection

**Table:**

Nonius KappaCCD diffractometer	2628 independent reflections
Radiation source: fine-focus sealed X-ray tube	1962 reflections with *I* > 2σ(*I*)
graphite	*R*_int_ = 0.071
φ scans, and ω scans with κ offsets	θ_max_ = 27.5°, θ_min_ = 3.5°
Absorption correction: multi-scan (*SADABS*; Bruker, 1998)	*h* = −14→14
*T*_min_ = 0.902, *T*_max_ = 0.902	*k* = −8→7
14084 measured reflections	*l* = −25→25

### Refinement

**Table:**

Refinement on *F*^2^	Secondary atom site location: difference Fourier map
Least-squares matrix: full	Hydrogen site location: inferred from neighbouring sites
*R*[*F*^2^ > 2σ(*F*^2^)] = 0.037	H-atom parameters constrained
*wR*(*F*^2^) = 0.098	*w* = 1/[σ^2^(*F*_o_^2^ + 0.3428*P*] where *P* = (*F*_o_^2^ + 2*F*_c_^2^)/3
*S* = 1.11	(Δ/σ)_max_ = 0.001
2628 reflections	Δρ_max_ = 0.55 e Å^−^^3^
156 parameters	Δρ_min_ = −0.97 e Å^−^^3^
0 restraints	Extinction correction: *SHELXL97* (Sheldrick, 2008), Fc^*^=kFc[1+0.001xFc^2^λ^3^/sin(2θ)]^-1/4^
Primary atom site location: structure-invariant direct methods	Extinction coefficient: 0.0084 (19)

### Special details

**Table:**

Geometry. All e.s.d.'s (except the e.s.d. in the dihedral angle between two l.s. planes) are estimated using the full covariance matrix. The cell e.s.d.'s are taken into account individually in the estimation of e.s.d.'s in distances, angles and torsion angles; correlations between e.s.d.'s in cell parameters are only used when they are defined by crystal symmetry. An approximate (isotropic) treatment of cell e.s.d.'s is used for estimating e.s.d.'s involving l.s. planes.

### Fractional atomic coordinates and isotropic or equivalent isotropic displacement parameters (Å^2^)

**Table:**

	*x*	*y*	*z*	*U*_iso_*/*U*_eq_	
Ni1	0.5	0.5	0.5	0.02521 (16)	
S1	0.75777 (6)	0.76611 (10)	0.53190 (4)	0.03283 (19)	
O5	0.22643 (18)	0.1935 (3)	0.18093 (9)	0.0384 (4)	
O1	0.62169 (17)	0.6626 (3)	0.46467 (10)	0.0387 (4)	
O2	0.47029 (16)	0.2626 (3)	0.42577 (9)	0.0307 (4)	
O3	0.32165 (16)	0.6292 (3)	0.40600 (9)	0.0316 (4)	
O4	0.1020 (2)	0.4798 (3)	0.15900 (11)	0.0478 (5)	
C6	0.2779 (2)	0.4022 (4)	0.29848 (13)	0.0252 (5)	
C3	0.1192 (3)	0.7190 (4)	0.28219 (15)	0.0378 (6)	
H3A	0.1101	0.8133	0.3173	0.057*	
H3B	0.0368	0.631	0.2527	0.057*	
H3C	0.1291	0.801	0.2446	0.057*	
C10	0.3256 (3)	0.0457 (4)	0.23083 (15)	0.0317 (6)	
C8	0.3859 (2)	0.2500 (4)	0.34964 (14)	0.0253 (5)	
C9	0.3994 (2)	0.0651 (4)	0.31075 (14)	0.0296 (5)	
H9	0.4616	−0.0426	0.3428	0.036*	
C5	0.2474 (2)	0.5807 (4)	0.33237 (13)	0.0265 (5)	
C2	0.7743 (3)	1.0036 (4)	0.48915 (18)	0.0469 (7)	
H2A	0.7626	0.9718	0.4382	0.07*	
H2B	0.8659	1.0642	0.5256	0.07*	
H2C	0.7034	1.1033	0.4805	0.07*	
C7	0.1958 (2)	0.3697 (4)	0.21184 (14)	0.0312 (6)	
C4	0.3357 (3)	−0.1273 (5)	0.18287 (17)	0.0470 (7)	
H4A	0.4053	−0.2289	0.2191	0.071*	
H4B	0.3623	−0.0679	0.1484	0.071*	
H4C	0.246	−0.1966	0.1501	0.071*	
C1	0.8954 (3)	0.6199 (5)	0.53594 (19)	0.0526 (8)	
H1A	0.8972	0.4776	0.5542	0.079*	
H1B	0.9841	0.6879	0.573	0.079*	
H1C	0.8792	0.6154	0.4828	0.079*	

### Atomic displacement parameters (Å^2^)

**Table:**

	*U*^11^	*U*^22^	*U*^33^	*U*^12^	*U*^13^	*U*^23^
Ni1	0.0190 (2)	0.0297 (3)	0.0197 (2)	0.00047 (17)	0.00610 (17)	−0.00322 (16)
S1	0.0280 (3)	0.0421 (4)	0.0247 (3)	−0.0061 (3)	0.0122 (3)	−0.0037 (3)
O5	0.0403 (10)	0.0450 (11)	0.0241 (9)	0.0070 (8)	0.0141 (8)	−0.0024 (7)
O1	0.0292 (9)	0.0534 (11)	0.0275 (9)	−0.0120 (8)	0.0119 (8)	−0.0051 (8)
O2	0.0245 (8)	0.0336 (9)	0.0212 (8)	0.0057 (7)	0.0046 (7)	−0.0021 (7)
O3	0.0257 (8)	0.0336 (10)	0.0260 (9)	0.0043 (7)	0.0083 (7)	−0.0040 (7)
O4	0.0461 (12)	0.0539 (12)	0.0237 (9)	0.0148 (10)	0.0068 (8)	0.0052 (8)
C6	0.0210 (11)	0.0284 (13)	0.0226 (11)	−0.0001 (10)	0.0099 (9)	0.0003 (9)
C3	0.0292 (13)	0.0375 (15)	0.0335 (13)	0.0092 (11)	0.0090 (11)	−0.0011 (11)
C10	0.0273 (13)	0.0352 (14)	0.0342 (13)	−0.0012 (11)	0.0179 (11)	−0.0038 (10)
C8	0.0209 (11)	0.0285 (12)	0.0255 (11)	−0.0034 (10)	0.0122 (9)	−0.0008 (9)
C9	0.0225 (12)	0.0309 (13)	0.0286 (13)	0.0003 (10)	0.0098 (10)	−0.0023 (10)
C5	0.0202 (11)	0.0286 (13)	0.0266 (12)	−0.0018 (10)	0.0104 (10)	0.0025 (9)
C2	0.0358 (15)	0.0372 (16)	0.0524 (17)	0.0002 (12)	0.0148 (13)	0.0047 (12)
C7	0.0292 (13)	0.0347 (14)	0.0264 (12)	0.0010 (11)	0.0132 (10)	0.0013 (10)
C4	0.0510 (17)	0.0524 (18)	0.0395 (15)	0.0046 (15)	0.0262 (14)	−0.0127 (13)
C1	0.0350 (15)	0.0460 (18)	0.0587 (19)	0.0043 (14)	0.0144 (14)	−0.0056 (14)

### Geometric parameters (Å, °)

**Table:**

Ni1—O2^i^	1.9849 (16)	C3—C5	1.507 (3)
Ni1—O2	1.9849 (16)	C3—H3A	0.96
Ni1—O3	2.0159 (15)	C3—H3B	0.96
Ni1—O3^i^	2.0159 (15)	C3—H3C	0.96
Ni1—O1^i^	2.1255 (18)	C10—C9	1.321 (3)
Ni1—O1	2.1255 (18)	C10—C4	1.487 (4)
S1—O1	1.5211 (17)	C8—C9	1.447 (3)
S1—C2	1.775 (3)	C9—H9	0.93
S1—C1	1.780 (3)	C2—H2A	0.96
O5—C10	1.371 (3)	C2—H2B	0.96
O5—C7	1.398 (3)	C2—H2C	0.96
O2—C8	1.262 (3)	C4—H4A	0.96
O3—C5	1.250 (3)	C4—H4B	0.96
O4—C7	1.215 (3)	C4—H4C	0.96
C6—C8	1.440 (3)	C1—H1A	0.96
C6—C7	1.441 (3)	C1—H1B	0.96
C6—C5	1.443 (3)	C1—H1C	0.96
			
O2^i^—Ni1—O2	180	C9—C10—C4	127.3 (2)
O2^i^—Ni1—O3	92.94 (6)	O5—C10—C4	111.1 (2)
O2—Ni1—O3	87.06 (6)	O2—C8—C6	125.9 (2)
O2^i^—Ni1—O3^i^	87.06 (6)	O2—C8—C9	116.6 (2)
O2—Ni1—O3^i^	92.94 (6)	C6—C8—C9	117.4 (2)
O3—Ni1—O3^i^	180.0000 (10)	C10—C9—C8	121.6 (2)
O2^i^—Ni1—O1^i^	89.72 (7)	C10—C9—H9	119.2
O2—Ni1—O1^i^	90.28 (7)	C8—C9—H9	119.2
O3—Ni1—O1^i^	89.31 (7)	O3—C5—C6	123.2 (2)
O3^i^—Ni1—O1^i^	90.69 (7)	O3—C5—C3	114.3 (2)
O2^i^—Ni1—O1	90.28 (7)	C6—C5—C3	122.51 (19)
O2—Ni1—O1	89.72 (7)	S1—C2—H2A	109.5
O3—Ni1—O1	90.69 (7)	S1—C2—H2B	109.5
O3^i^—Ni1—O1	89.31 (7)	H2A—C2—H2B	109.5
O1^i^—Ni1—O1	180	S1—C2—H2C	109.5
O1—S1—C2	105.60 (12)	H2A—C2—H2C	109.5
O1—S1—C1	105.41 (12)	H2B—C2—H2C	109.5
C2—S1—C1	97.65 (15)	O4—C7—O5	112.9 (2)
C10—O5—C7	121.85 (18)	O4—C7—C6	128.7 (2)
S1—O1—Ni1	116.97 (9)	O5—C7—C6	118.4 (2)
C8—O2—Ni1	129.32 (15)	C10—C4—H4A	109.5
C5—O3—Ni1	131.49 (15)	C10—C4—H4B	109.5
C8—C6—C7	118.8 (2)	H4A—C4—H4B	109.5
C8—C6—C5	121.45 (19)	C10—C4—H4C	109.5
C7—C6—C5	119.74 (19)	H4A—C4—H4C	109.5
C5—C3—H3A	109.5	H4B—C4—H4C	109.5
C5—C3—H3B	109.5	S1—C1—H1A	109.5
H3A—C3—H3B	109.5	S1—C1—H1B	109.5
C5—C3—H3C	109.5	H1A—C1—H1B	109.5
H3A—C3—H3C	109.5	S1—C1—H1C	109.5
H3B—C3—H3C	109.5	H1A—C1—H1C	109.5
C9—C10—O5	121.6 (2)	H1B—C1—H1C	109.5

Symmetry codes: (i) −*x*+1, −*y*+1, −*z*+1.

### Hydrogen-bond geometry (Å, °)

**Table:**

*D*—H···*A*	*D*—H	H···*A*	*D*···*A*	*D*—H···*A*
C1—H1B···O4^ii^	0.96	2.55	3.384 (4)	145
C2—H2B···O4^ii^	0.96	2.53	3.370 (4)	146
C2—H2C···O2^iii^	0.96	2.46	3.378 (4)	160

Symmetry codes: (ii) *x*+1, −*y*+3/2, *z*+1/2; (iii) *x*, *y*+1, *z*.
